# Impact of post-traumatic stress disorder symptoms, childhood adversities and stressful life events on depressive and anxiety symptoms: insights from the UK Biobank

**DOI:** 10.3389/fpsyt.2025.1488320

**Published:** 2025-03-21

**Authors:** Qianshu Ma, Min Xie, Elyse Llamocca, Yupeng Luo, Liling Xiao, Yiguo Tang, Shiwan Tao, Yulu Wu, Yunqi Huang, Yubing Yin, Yunjia Liu, Siyi Liu, Renhao Deng, Chunxia Qiao, Menghan Wei, Yang Chen, Jia Cai, Hongsheng Gui, Qiang Wang

**Affiliations:** ^1^ West China Brain Research Center, West China Hospital of Sichuan University, Chengdu, Sichuan, China; ^2^ Sichuan Clinical Medical Research Center for Mental Disorders, Mental Health Center, West China Hospital, Sichuan University, Chengdu, China; ^3^ Center for Health Policy and Health Services Research, Henry Ford Health, Detroit, MI, United States; ^4^ Behavioral Health Services and Psychiatry Research, Henry Ford Health, Detroit, MI, United States; ^5^ West China Hospital, Sichuan University, Chengdu, China

**Keywords:** childhood adversities, stress life events, post-traumatic stress disorder symptoms, depressive symptoms, anxiety symptoms, UK Biobank

## Abstract

**Background:**

Childhood adversities (CAs) and stressful life events (SLEs) are linked to depressive, anxiety, and PTSD symptoms. However, their interrelationships are not well studied. We aimed to examine the potential role of PTSD symptoms as risk factors for both outcomes, test the stress sensitization hypothesis, and investigate the pathways linking CAs, stressful life events (SLEs) and PTSD symptoms, and depressive and anxiety symptoms.

**Methods:**

We conducted a cross-sectional study using data from adult participants at baseline (2006-2010) and online follow-up (2016) in the UK Biobank. Data analysis was performed from February 24, 2023, to July 12, 2023. Linear regression and serial mediation analyses were performed.

**Results:**

PTSD symptoms was significantly associated with depressive (β = 0.567, p<.001) and anxiety symptoms (β = 0.558, p<.001). The interaction between CAs and SLEs was still significantly associated with depressive symptoms when accounting for those of PTSD as covariates (β = 0.017, p<.001), but not for anxiety symptoms. The serial mediation analyses revealed that SLEs and PTSD symptoms were both significant sequential mediators between CAs and symptoms of depression and anxiety (proportion mediated: 75.14% and 84.27%, respectively, p< 0.05).

**Conclusions:**

Our study provided further evidence for stress sensitization hypothesis only among participants with depressive symptoms and found that SLEs and PSTD symptoms partly mediated the association between CAs and depressive and anxiety symptoms. These findings may provide new evidence to better understand the pathogenesis of depression and anxiety and will help to guide future prevention and intervention for both diseases.

## 1 Introduction

Depression and anxiety are ubiquitous mental health disorders with high global prevalence. According to the World Health Organization survey conducted in 2019, an estimated 280 million individuals were affected by depression worldwide, while approximately 301 million individuals suffered from anxiety disorders (1). Santomauro et al. found that the prevalence of depression and anxiety has continued to increase since 2020, partially due to the COVID-19 pandemic (2). These disorders also pose considerable economic challenges, with depression as the second leading specific cause of Years Lived with Disability (YLDs) (3) and anxiety disorders ranking as the sixth leading cause of disability (4).

The pathogenesis of depression and anxiety is intricate and multifactorial, with both biopsychosocial and environmental factors playing important roles. Childhood adversities (CAs) are associated with increased risk for mental illnesses (5–7) through biological mechanisms (8–10). Similarly, stressful life events (SLEs) have robust relationships with depression (11) and anxiety (12). Nevertheless, individual CAs and adult SLEs cannot completely account for the occurrence and development of depression and anxiety. Post-traumatic stress disorder (PTSD) is a psychiatric disease associated with stress after experiencing traumatic events. Its key features include avoidance, emotional disturbances, hyperarousal, and intrusive recollections. PTSD is highly comorbid with depression and anxiety (13). Recently, a longitudinal study suggested that PTSD could be an important risk factor for depression and anxiety (14). Since PTSD, depression, and anxiety are highly interlinked, the associations between these diagnoses need to be further investigated.

Prior research has suggested that CAs and SLEs contribute independently to the onset of depression (15, 16) and anxiety (17), while the interplay between the two is also associated with those mood disorders. The mechanism underlying the association could potentially be explained by the stress sensitization hypothesis (18), which states that individuals experiencing CAs would be easier to have depressive and anxiety reactions to stress. However, the stress sensitization hypothesis has been tested in different populations with inconsistent results. A longitudinal randomized controlled trial reported that compared to those without childhood adversity in childhood, individuals with prolonged adversity in preadolescence would have higher externalizing problems when experiencing stressful life events in adolescence (19). In contrast, another recent longitudinal study found that the association between early life adversity status and depressive symptoms was not modified by the totality of recent SLEs in older adults (20). In addition to the inconsistent results across different studies, current studies also lack the incorporation of multiple covariates and larger sample sizes.

To elucidate the pathway from CAs to depression, and anxiety, we hypothesize that CAs may lead to depressive and anxiety symptoms by increasing PTSD symptoms following SLEs. Previous findings have illustrated that CAs were significantly associated with recent stress in adulthood (21). A cross-sectional study found that stress in adulthood mediated associations between CAs and depression and anxiety (22). SLEs were found to have significant direct effects on PTSD symptoms among adolescents (23). Several studies have shown that PTSD symptoms play a potential mediating role in the relationship between CAs and depression (24) and anxiety (25). The existing literature is limited by study design, as most research consists of small-scale studies (26, 27). We intended to address these gaps in the literature by testing our hypothesized pathways using data from the UK Biobank (UKB), a large-scale population-based cohort (28). In the UKB, the information on SLEs was collected before that of PTSD symptoms. Due to the order of collection time of the variables, we put the mediators in the SLEs-PTSD symptoms order in the hypothesized serial mediating model.

In this study, we aim to better understand the linkage between CAs, SLEs, PTSD, and depression and anxiety symptoms by: (1) investigating whether PTSD symptoms are associated with depressive and anxiety symptoms; (2) testing if the association between CAs and depressive and anxiety symptoms is still moderated by SLEs adjusting for more covariates in the general population; and (3) exploring the direct and indirect paths from CAs to depressive and anxiety symptoms via exposure to a cumulative number of SLEs and PTSD symptoms.

## 2 Methods

### 2.1 Participants and data source

The UK Biobank (UKB) cohort was assembled across 22 assessment centres in the UK between 2006 and 2010, recruiting over 500,000 participants aged from 38 to 72. Among them, 157,198 completed questionnaires of interest in the online follow-up mental health survey, including the 9-item Patient Health Questionnaire (PHQ-9), the 7-item General Anxiety Disorder (GAD-7), and the 6-item PTSD checklist (PCL-6) (**Supplementary Figure S1**).

This was a retrospective cohort study. We excluded participants with missing values related to CAs (n = 350,089), those who did not complete the online follow-up mental health survey (n= 55), and those who had missing values on demographic variables of interest (n = 1,265) (**Supplementary Figure S1**).

All participants in the UKB cohort have provided written consent. Full ethical approval was given by the National Research Ethics Service (NHS) (16/NW/0274). Data access for this project was granted by application 86920. Our study was supported by the Biomedical Research Ethics Committee of West China Hospital (2019-1171). Our study followed the Strengthening the Reporting of Observational Studies in Epidemiology (STROBE) reporting guidelines.

### 2.2 Assessment of CAs and SLEs

**Figure 1:**
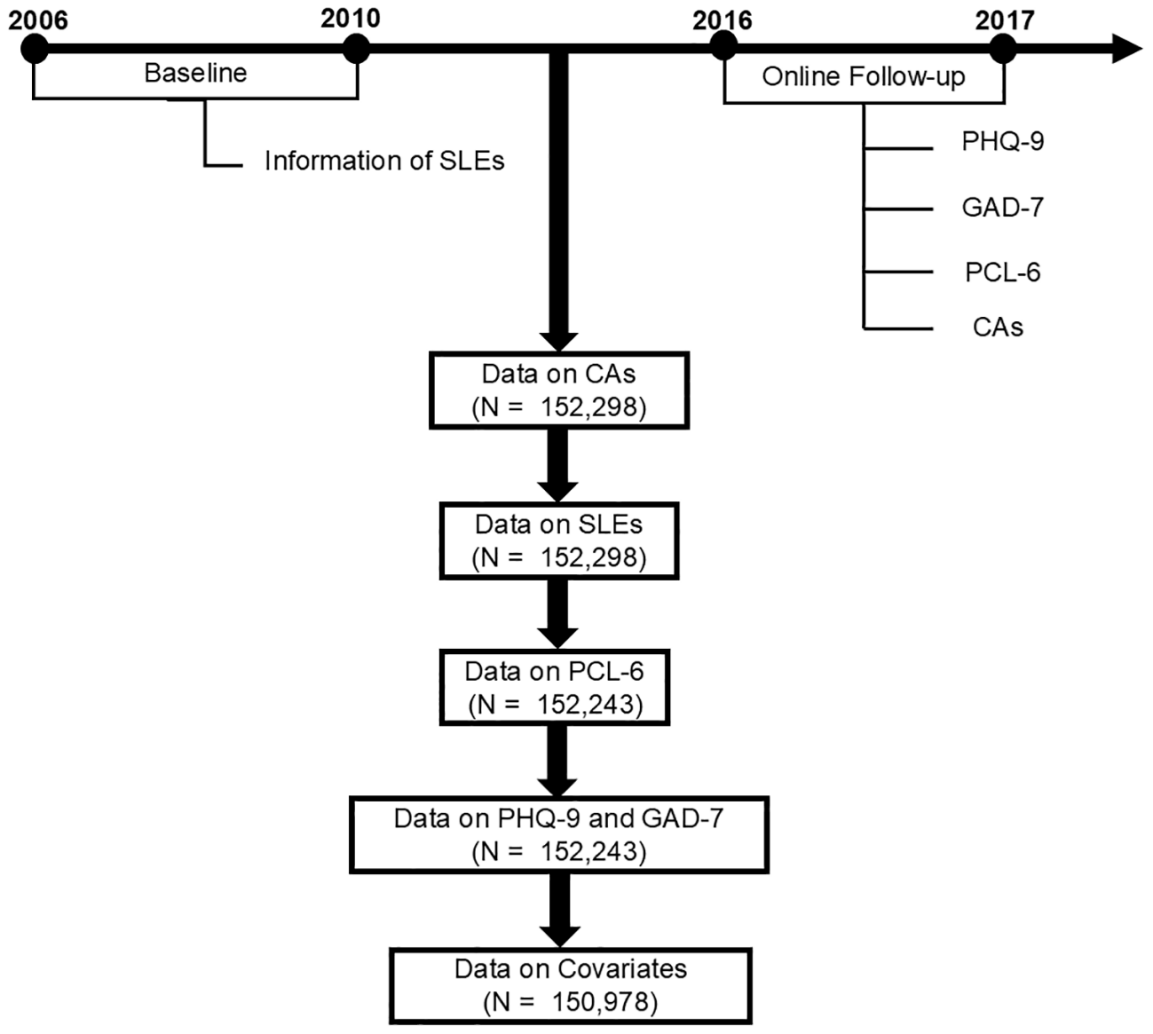
Flowchart. CAs, childhood adversities; GAD-7, the 7-item General Anxiety Disorder; PHQ-9, the 9-item Patient Health Questionnaire; PCL-6, the 6-item PTSD checklist; SLEs, stressful life events.

Information on CAs was collected in the online follow-up survey between 2016 and 2017 (**Figure 1**). CAs were assessed using the Childhood Trauma Screener, a shortened version of Childhood Trauma Questionnaires, which consisted of 5 questions regarding abuse and neglect. Responses ranged from “never true =0” to “very often true =4”. Answers of “prefer not to answer” were treated as missing data. We reverse-coded two questions about neglect so that all questions were scored from 0 to 4, with higher values indicating more frequent neglect. We summed responses from all 5 questions to get a total score for analyses (possible range of total scores: 0 to 20), with higher scores representing higher levels of childhood trauma. The screener is a relatively reliable tool that has been used in many epidemiological studies. The details of the screener and data fields are shown in **Supplementary Table S1** in the **Supplementary**.

Information on SLEs was collected at baseline between 2006 and 2010 (**Figure 1**) through self-report of illness, injury, bereavement, and stress (Data-Field 6145). Participants were asked whether they had experienced six possible SLEs in the previous two years: 1) “serious illness, injury or assault to yourself”, 2) “serious illness, injury or assault of a close relative”, 3) “death of a close relative”, 4) “death of a spouse or partner”, 5) “marital separation/divorce”, and 6) “financial difficulties”. The total number of SLEs was calculated by summing the number of SLEs that a given participant reported experiencing, with a higher number representing a higher level of stress.

### 2.3 Depressive, anxiety, and PTSD symptoms

In 2016-2017, all the UKB participants at baseline received a link to the Mental Health Questionnaires (MHQ) (29) via emails, and about 31% of the UKB cohort responded in time. Sourced from the MHQ, three questionnaires including PHQ-9, GAD-7, and PCL-6 were used to assess depressive and anxiety symptoms over the past two weeks and PTSD symptoms in the past month, respectively. The total score of each of the three questionnaires (PHQ-9, 0-27; GAD-7, 0-21; PCL-6, 0-24) separately represented one continuous outcome variable. The higher score suggested more severe depression. Previous studies have shown that PHQ-9 (30), GAD-7 (31), and PCL-6 (32) are scales with strong internal consistency and high sensitivity in screening major depressive disorders, generalized anxiety disorders, and PTSD.

### 2.4 Covariates

In this study, sociodemographic variables included age, sex, race, education level, and socioeconomic status. Original ethnicity in UKB was coded as White, Mixed, Asian, Black, and others. Since the sample was relatively homogeneous in terms of race, we defined race as a binary variable, i.e., White, or non-White. We identified the socioeconomic status based on the Townsend deprivation index (TDI), an index that reflects the percentage of unemployed and the percentage of overcrowded households, not owner-occupied, or do not own a car or van. The education level and TDI were significantly associated with depressive and anxiety symptoms according to previous findings (33–35). All the covariates were extracted from self-reported surveys collected at baseline (2006-2010).

### 2.5 Statistical analyses

All analyses were performed with R (version 4.3.1). R package (“Tableone”) was used to summarize participants’ sociodemographic characteristics, which were described with a mean and standard deviation (SD) for continuous variables and count (%) for categorical variables, respectively. Spearman correlation analysis was conducted to calculate the correlation between exposures and outcomes using the R package (“bruceR”). The false-discovery rate (FDR) correction was performed to adjust for multiple testing in the correlation analyses. We fit the following linear regression models. In these models, Y is the total score of PHQ-9/GAD-7. X_1_ is the total score of PCL-6. X_2_ is the total score of CAs and X_3_ is the total number of SLEs. Covariates include age, sex, race, education and TDI.

Model 1: The association between PTSD symptoms and depressive and anxiety symptoms.


Y~β1X1+Covariates

Model 2: The interaction between CAs and SLEs as a predictor of depressive and anxiety symptoms.


Y~β2X2+β3X3+β4X2*X3+Covariates

Model 3: The interaction between CAs and SLEs as a predictor of depressive and anxiety symptoms, when adding PTSD symptoms as covariates.


Y~β1X1+β2X2+β3X3+β4X2*X3+Covariates

Serial multiple mediation models were adopted to assess the relationships among our exposures and outcomes of interest and the potential mediating effects of SLEs and PTSD symptoms on the associations between CAs and depressive and anxiety symptoms. This was done using the R package (“bruceR”). We conducted the bootstrapping analyses 1,000 times to test the significance of the mediating effect. For pathways in serial multiple mediation analyses, we calculated estimated direct, specific indirect, total indirect, and total mediation effects.

In additional analyses, in order to investigate the generalisability of the sample after being excluded based on missing values, we compared the baseline characteristics of participants excluded because of missing data and participants included in our study. We used chi-test to compare the distribution of gender, education level and race between these two groups. T-test was used to compare the mean of age and TDI of the two groups.

## 3 Results

### 3.1 Sample characteristics

The total sample included 150,978 participants. As shown in **Table 1**, participants in this study tended to be female (56.3%), white (91.0%), and have a university or college degree (63.8%). The mean age was 55.91 years old (SD: 7.74) and the mean TDI was -1.72 (SD: 2.83). Nearly three-fifths of the sample (59.2%) had experienced childhood adversities, and 42.3% reported at least one stressful life event.

**Table 1:** Baseline Characteristics of the Participants.

Characteristics	Participants (N=150,978)	Minimum value	Maximum value
Covariates			
Sex (Women)	85,033 (56.3%)	/	/
Age	55.91 ± 7.74	38	72
Townsend deprivation index	-1.72 ± 2.83	-6.2583	11.0013
Education (With a university degree)	96,297 (63.8%)	/	/
Race (White)	137,442 (91.0%)	/	/
Exposures			
Number of SLEs	0.56 ± 0.75	0	4
Number of SLEs >=1	63,894 (42.3%)	/	/
Total score of CAs	1.75 ± 2.40	0	20
Total score of CAs >= 1	89,395 (59.2%)	/	/
PCL-6 Total score	2.95 ± 3.05	0	24
Outcomes			
PHQ-9 Total score	2.69 ± 3.64	0	27
GAD-7 Total score	2.14 ± 3.37	0	21

In **Table 1**, “/” represents binary variables in the minimum and maximum columns; The Mean and SD are presented in the form of “Mean ± SD”; CAs, childhood adversities; GAD-7, the 7-item General Anxiety Disorder; PHQ-9, the 9-item Patient Health Questionnaire; PCL-6, the 6-item PTSD checklist; SLEs, stressful life events; SD, Standard Deviation.

### 3.2 Correlation analysis

Pairwise correlations among our exposures (total CA score, total SLEs experienced, and total PCL-6 score) and outcomes (total PHQ-9 and GAD-7 scores) are shown in **Supplementary Figure S1**. After FDR correction, all pairs of variables were significantly and positively correlated (*p<*.001). The correlation between CAs and depressive and anxiety symptoms was 0.183 and 0.170, respectively; and the correlation between SLEs and depressive and anxiety symptoms was 0.105 and 0.122, respectively. PTSD symptoms and depressive and anxiety symptoms were highly correlated (0.466 and 0.483).

### 3.3 Linear regression

We conducted multivariable linear regression to find whether PTSD symptoms are associated with depressive and anxiety symptoms and to investigate the effect of the interaction between CAs and SLEs on the outcomes.

Model 1: The association between PTSD symptoms and depressive and anxiety symptoms.

After adjustment for covariates, PTSD symptoms were significantly positively associated with depressive (β = 0.567; 95% CI, 0.563 - 0.572), and anxiety symptoms (β = 0.558; 95% CI, 0.553 - 0.562) (**Table 2**).

**Table 2:** Results of linear regression models.

Independents variables	Depressive symptoms				Anxiety symptoms			
	β(95%CI)	SE	*P* value	R^2^	β(95%CI)	SE	*P* value	R^2^
Model 1				0.349				0.332
PTSD symptoms	0.567 (0.563,0.572)	0.003	<.001		0.558 (0.553,0.562)	0.002	<.001	
Model 2				0.102				0.079
CAs	0.195 (0.185,0.205)	0.004	<.001		0.177 (0.168,0.186)	0.005	<.001	
SLEs	0.085 (0.056, 0.114)	0.015	<.001		0.065 (0.038,0.092)	0.014	<.001	
CAs * SLEs	0.049 (0.041,0.057)	0.004	<.001		0.036 (0.028,0.044)	0.004	<.001	
Model 3				0.355				0.334
CAs	0.048 (0.004,0.006)	0.004	<.001		0.029 (0.022,0.037)	0.004	<.001	
SLEs	0.038 (0.001,0.006)	0.013	<.001		0.018 (-0.006,0.041)	0.012	<.001	
PTSD symptoms	0.542 (0.540,0.550)	0.003	<.001		0.545 (0.0540,0.055)	0.003	<.001	
CAs * SLEs	0.017 (0.010,0.020)	0.004	<.001		0.004 (-0.002,0.011)	0.003	0.190	

CAs, childhood adversities; SE, standard error; SLEs, stressful life events.

Model 2: The association of the interaction between CAs and SLEs with depressive and anxiety symptoms.

**Figure 2:**
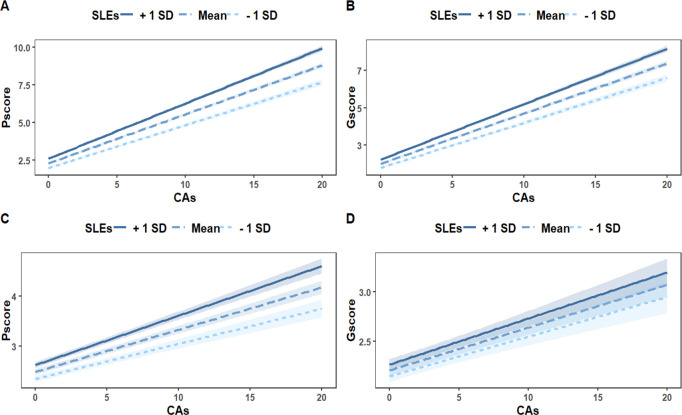
The moderation effect of SLEs on the relationship between CAs and Depressive and Anxiety Symptoms. **(A, B)** showed the association of the interaction between CAs and SLEs with depressive **(A)** and anxiety symptoms **(B)**. **(C, D)** showed the association of the interaction between CAs and SLEs with depressive **(C)** and anxiety symptoms **(D)** when accounting for PTSD symptoms. CAs, childhood adversities; Gscore, the total score of 7-item General Anxiety Disorder; Pscore, the total score of the 9-item Patient Health Questionnaire; SLEs, stressful life events.

After adjustment for covariates, CAs and SLEs were significantly positively associated with depressive (β_CAs_ = 0.195; 95% CI, 0.185 - 0.205; β_SLEs_ = 0.085; 95% CI, 0.056 - 0.114) and anxiety symptoms (β_CAs_ = 0.177; 95% CI, 0.168 - 0.186; β_SLEs_ = 0.065; 95% CI, 0.038 - 0.092). The interaction between CAs and SLEs significantly predicted depressive (β = 0.049; 95% CI, 0.041 - 0.057) and anxiety symptoms (β = 0.036; 95% CI, 0.028 - 0.044), respectively (**Table 2**). In other words, individuals with CAs were more likely to develop depressive and anxiety symptoms when facing SLEs in adulthood compared to those without CAs (**Figure 2A, B**).

Model 3: The association of the interaction between CAs and SLEs with depressive and anxiety symptoms, when accounting for PTSD symptoms.

The association between CAs and depressive symptoms was still significantly moderated by SLEs even after accounting for PTSD symptoms (β = 0.017; 95% CI, 0.010 - 0.020) (**Figure 2C**, **Table 2**). In the contract, SLEs did not significantly moderate the association between CAs and anxiety symptoms after accounting for PTSD symptoms (β = 0.004; 95% CI, -0.002 - 0.011) (**Figure 2D**, **Table 2**).

### 3.4 Mediation analyses

**Figure 3:**
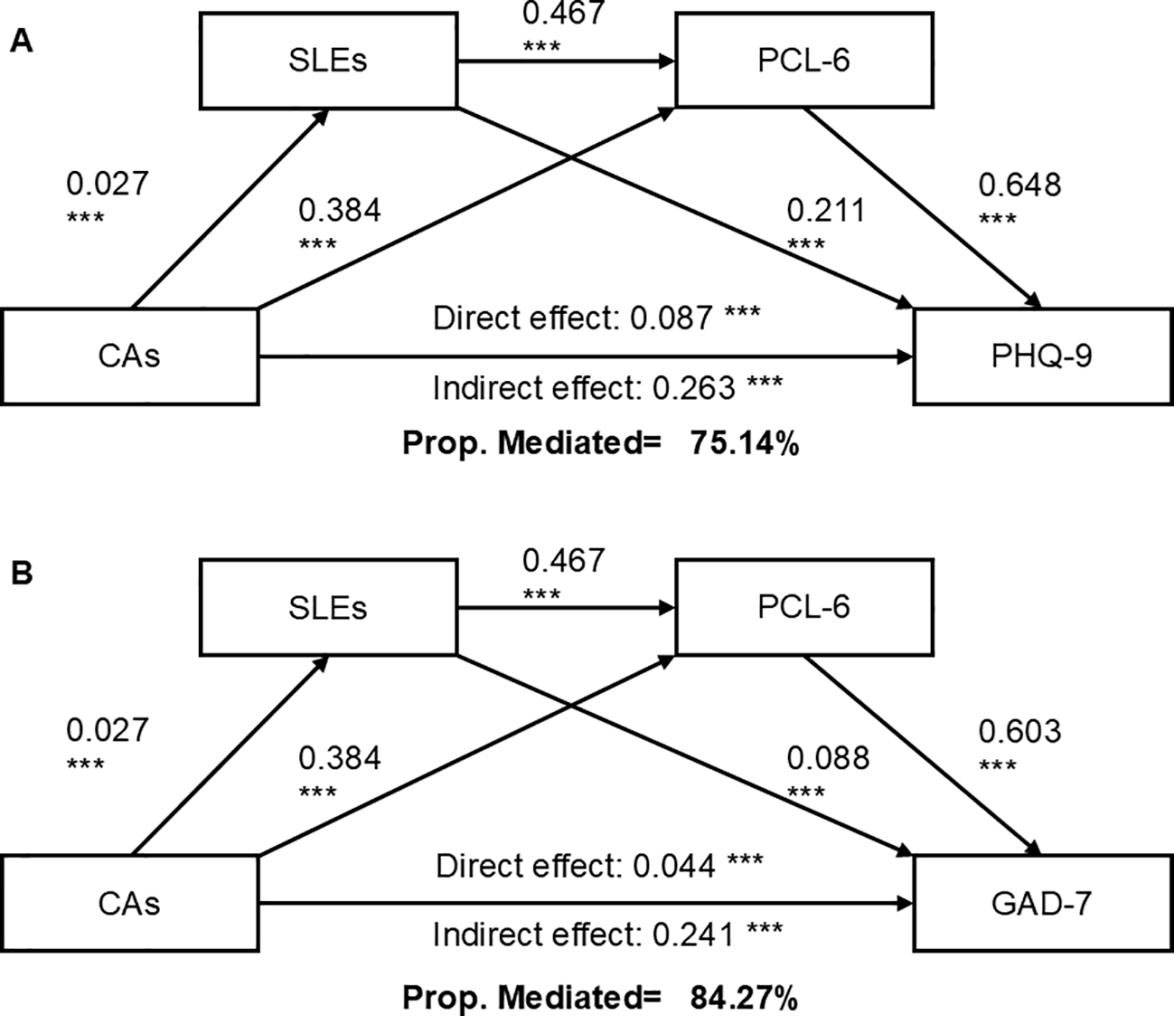
Serial mediation effects of SLEs and PTSD Symptoms between Childhood adversities and Depressive **(A)** and Anxiety Symptoms **(B)**. ***: P<.001; CAs, childhood adversities; GAD-7, the 7-item General Anxiety Disorder; PHQ-9, the 9-item Patient Health Questionnaire; PCL-6, the 6-item PTSD checklist; Prop. Mediated, Mediated Proportion; SLEs, stressful life events.

Serial multiple mediation analyses were performed to evaluate the potential mediation effects of SLEs and PTSD symptoms on the association between CAs and depressive and anxiety symptoms. All the paths of these two models were significant (*p<*.001). CAs indirectly and partly predicted depressive and anxiety symptoms, with total indirect effects of 0.263 and 0.241, respectively. As shown in **Figure 3A**, the SLEs-PTSD symptoms path serially mediated the association between CAs and depressive symptoms, accounting for 75.14% of the observed association. Similarly, the SLEs-PTSD symptoms path also serially mediated the association between CAs and anxiety symptoms, with 84.27% of the association mediated (**Figure 3B**). **Supplementary Table S3** in the **Supplementary Material** showed that both models exhibited acceptable goodness of fit, with R^2^ of 0.354 and 0.334, respectively. Finally, **Supplementary Table S4** shows that the series mediating effects of the two paths (CAs → Stressful life events → PCL-6 → PHQ-9 and CAs → Stressful life events → PCL-6 → GAD-7) were both significant (β = 0.008; 95% CI, 0.007 - 0.009; β = 0.007; 95% CI, 0.007 - 0.008). More details can be found in the online **Supplementary Material**.

### 3.5 Additional analyses

We found that participants who were excluded because of missing values were more likely to be older males with lower social status and education levels and less likely to be white compared with the total population included in our study (**Supplementary Table S2**).

## 4 Discussion

Using population-based cohort data from the UKB, the aim of our study was to assess the relationship between CAs, SLEs, PTSD, depression, and anxiety at the symptom level. Our studies had three findings. First, PTSD symptoms are significantly associated with both depressive and anxiety symptoms. Secondly, SLEs still moderated the association between CAs and depressive symptoms after accounting for PTSD symptoms as covariates, but not for that between CAs and anxiety symptoms. Finally, SLEs and PSTD symptoms partly and sequentially mediated the association between CAs and depressive and anxiety symptoms.

Our first finding aligns with recent longitudinal studies (36, 37) corroborating the close relationship between PTSD symptoms and depressive and anxiety symptoms. Apart from observational epidemiological research, there is some genetic and neurodevelopmental evidence that can further support our findings. Zhang F et al. have unveiled that genetically determined PTSD confers a causal effect on broad depression phenotype (38). Recent research has highlighted common structural changes in the brain between anxiety disorders and PTSD, revealing that diminished integrity of the uncinate fasciculus serves as a shared neural basis for both conditions (39, 40).

CAs and SLEs were both associated with symptoms of depression and anxiety in this study, which was consistent with previous research (12, 41, 42). Moreover, our findings may provide further evidence for the stress sensitization hypothesis (18), which posits that the effects of child adversity on depressive symptoms in adults may be moderated by stressful life events. Results were in line with previous studies that reported that stress in adulthood positively moderated the relationship between CAs and major depressive disorder (11) or depressive symptoms (43). However, after including PTSD symptoms as a covariate in the model, only the association between CAs and depressive symptoms was significantly moderated by SLEs, while no significant result was found in the association between CAs and anxiety symptoms. This null finding was consistent with a previous study among students exposed to the war in Uganda that found no significant association between anxiety symptoms and the interaction between stressful war experience and childhood maltreatment (44). However, our finding contradicted the result of another study showing that adult stress significantly moderated the association between childhood adversities and general anxiety disorder (GAD) in U.S. soldiers (45). One possible explanation is that PTSD, which many U.S. soldiers suffer from (46), may play a more important role in the association between CAs, SLEs, and GAD, but that study did not consider PTSD as a covariate. Additionally, although depression and anxiety have considerable overlap in symptoms (47) and risk factors (17), they are essentially two similar but different diseases in terms of genetic (48) and neuroimaging (49) mechanisms. The null finding of this study may be able to supplement the differences between the two from an epidemiological perspective. Continued studies are needed to investigate whether the association between CAs and anxiety symptoms is modified by recent stress.

Furthermore, we revealed the pathway connecting CAs and depressive or anxiety symptoms through sequential mediating patterns of the SLEs and PTSD symptoms. In the pathway of CAs-SLEs-PTSD, CAs have been established as a potent predisposing risk factor for stress-related psychopathology following subsequent trauma exposure in adulthood. In other words, adversity in early childhood increases the chance of adulthood exposure to later psychological trauma (50) leading to the development of stress-related psychopathology, including PTSD, depression, and anxiety (51), therefore contributing to stress susceptibility (52). Several independent lines of research support this scenario. Emerging evidence suggests that CAs increase vulnerability to the effects of later SLEs (19). Moreover, McLaughlin et al. found that past-year stressful life events were associated with an increased risk of perceived stress, PTSD, major depression, and anxiety disorders (11). In addition, prior research has found PTSD symptoms mediated the association between childhood maltreatment and depressive symptoms (53). The cumulative evidence from these studies suggests that CAs increase depression as well as anxiety through SLEs and PTSD symptoms.

Lastly, our findings revealed a difference in the percentage of mediation between the two pathways—through SLE and through PTSD—from CAs to depressive/anxiety symptoms. There might be two reasons behind this phenomenon. Firstly, there are common genetic factors among PTSD symptoms, depressive and anxiety symptoms (54). This suggests that PTSD symptoms may have a more direct or robust influence on depressive and anxiety outcomes compared to SLEs. Consequently, the mediation pathway through PTSD captures a larger proportion of the overall association between CAs and depressive/anxiety symptoms. This stronger link may partly explain why the mediation effect via PTSD is considerably higher. Another contributing factor may be the nature of the assessments used. The evaluation of PTSD symptoms, as well as depressive and anxiety symptoms, relies on subjective self-report measures, which might capture subtle nuances and internal states more comprehensively (30–32). In contrast, the assessment of SLEs is based on more objective criteria. This difference in measurement approach could lead to variations in how accurately and consistently these constructs are captured, potentially influencing the observed mediation percentages. The subjectivity of symptom assessments may result in stronger associations and higher mediation percentages for the PTSD pathway compared to the more objective SLE measures.

### 4.1 Strengths and limitations

Our study has several strengths, including validating potential risk factors using large-scale cohort data obtained from a general population sample and investigating diverse moderators and mediators of the relationship between CAs and mental health distress such as depressive and anxiety symptoms (55). However, our study also has several limitations. First, although we quantified the cumulative number of stressful life events, these measures do not necessarily capture perceived stress or biological responses to stress. Second, the mean scores of PHQ-9, GAD-7 and PCL-6 were relatively low, because the UKB is a population-based cohort instead of a disease-based cohort, with most participants having relatively good mental status. Similarly, the time lag between the time points when collecting data about SLEs and psychiatric symptoms was relatively long (about 6 years) as the UKB was not designed as a typical longitudinal cohort. Also, PTSD symptoms were measured at the same time as the depressive and anxiety symptoms, so further investigations into causality are needed to support our findings. Lastly, our findings may lack generalizability due to volunteer bias. Since the majority of individuals included in our study voluntarily participated in the online follow-up, those who were excluded were more likely to be older males with lower social status and education levels and less likely to be white compared to the total study population. Therefore, our findings should be interpreted with caution.

## 5 Conclusion

In summary, we identify potential risk factors for depressive and anxiety symptoms in a large-scale general population sample, by finding that PTSD symptoms positively associated both depressive and anxiety symptoms. Additionally, our results lend support to the stress sensitization hypothesis, suggesting that exposure to CAs amplifies the vulnerability to depressive symptoms in the context of later stressful life events. What is more, we depicted new mediation pathways from CAs to emotional distress including depressive and anxiety symptoms through SLEs and PTSD symptoms. Our study contributes to the growing body of literature examining the multifaceted relationship between stress, mental health symptoms, and underlying risk factors, providing a nuanced understanding that could inform both preventive and therapeutic strategies.

## Data Availability

The original contributions presented in the study are included in the article/**Supplementary Material**. Further inquiries can be directed to the corresponding authors.
