# Supplementary material for: Impact of post-traumatic stress disorder symptoms, childhood adversities and stressful life events on depressive and anxiety symptoms: insights from the UK Biobank
Source: Front Psychiatry. 2025 Mar 21;16:1488320. doi: 10.3389/fpsyt.2025.1488320 (PMC11969116; doi:10.3389/fpsyt.2025.1488320)
Supplement: Supplementary file 2 [file DataSheet2.docx]

# Supplementary Materials: Impact of Post-Traumatic Stress Disorder Symptoms, Childhood Adversities, and Stressful Life Events on Depressive and Anxiety Symptoms: Insights from the UK Biobank

**Table S1. Questions, Data-Field IDs and Responses for Variables Included in the Childhood Adversities in the UKB**

| Data-Field ID | Questions | | Responses |
| --- | --- | --- | --- |
| 20489 | Emotional neglect | Felt loved as a child | 0, Never true; 1, Rarely true; 2, Sometimes true; 3, Often;  4, Very often true |
| 20491 | Physical neglect | Someone to take to doctor when needed as a child | 0, Never true; 1, Rarely true; 2, Sometimes true; 3, Often;  4, Very often true |
| 20487 | Emotional abuse | Felt hated by family member as a child | 0, Never true; 1, Rarely true; 2, Sometimes true; 3, Often;  4, Very often true |
| 20488 | Physical abuse | Physically abused by family as a child | 0, Never true; 1, Rarely true; 2, Sometimes true; 3, Often;  4, Very often true |
| 20490 | Sexual abuse | Sexually molested as a child | 0, Never true; 1, Rarely true; 2, Sometimes true; 3, Often;  4, Very often true |

**Table S2. Baseline Characteristics of Participants Excluded Because of Missing Data and the Participants Included in Our Study**

|  |  | Participants exclused  (N = 351,409) | Participants included  (N = 150,978) | *P* value |
| --- | --- | --- | --- | --- |
| Sex (%) | Women | 188,277 (53.6) | 85,033 (56.3) | <.001 |
|  | Men | 163,132 (46.4) | 65,945 (43.7) | |
| Age (mean (SD)) |  | 56.79 (8.23) | 55.91 (7.74) | <.001 |
| TDI (mean (SD)) |  | -1.11 (3.19) | -1.72 (2.83) | <.001 |
| Education (%) | With university degree | 190,766 (54.9) | 96,297 (63.8) | <.001 |
| Race (%) | White | 305,053 (87.4) | 137,442 (91.0) | <.001 |
|  | Non-white | 46,356(12.6) | 13,536 (9.0) |  |

**Table S3. Information of the Two Paths from CAs to Depressive and Anxiety Symptoms**

|  | **β** | **SE** | ***P* value** | **R^2^** |
| --- | --- | --- | --- | --- |
| **PATH: CAs→ SLEs→ PCL-6→ PHQ-9** | | | | |
| Dependent variable: SLEs | | | | |
| CAs | 0.027 *** | .001 | <.001 | 0.025 |
| Dependent variable: PCL-6 | | | | |
| CAs | 0.384*** | 0.003 | <.001 | 0.140 |
| SLEs | 0.467*** | 0.01 | <.001 |  |
| Dependent variable: PHQ-9 | | | | |
| CAs | 0.087*** | 0.003 | <.001 | 0.354 |
| SLEs | 0.221*** | 0.01 | <.001 |  |
| PCL-6 | 0.648*** | 0.003 | <.001 |  |
| **PATH: CAs→ SLEs→ PCL-6→ GAD-7** | | | | |
| Dependent variable: SLEs | | | | |
| CAs | 0.027 *** | .001 | <.001 | 0.025 |
| Dependent variable: PCL-6 | | | | |
| CAs | 0.384*** | 0.003 | <.001 | 0.14 |
| SLEs | 0.467*** | 0.01 | <.001 |  |
| Dependent variable: GAD-7 | | | | |
| CAs | 0.044*** | 0.003 | <.001 | 0.334 |
| SLEs | 0.088*** | 0.01 | <.001 |  |
| PCL-6 | 0.603*** | 0.003 | <.001 |  |

*Note: CAs: childhood adversities; SLEs: stressful life events; SE: standard error; PHQ-9: the 9-item Patient Health Questionnaire, in this table it refers to the total score of PHQ-9; GAD-7: the 7-item General Anxiety Disorder, in this table it refers to the total score of GAD-7; PCL-6: the 6-item PTSD checklist, in this table it refers to the total score of PCL-6*

**Table S4. Results of Mediation Analyses**

| Indirect effects | | | |
| --- | --- | --- | --- |
| β | SE | LLCI | ULCI |
| Dependent variable: PHQ-9 | | | |
| CAs→ SLEs→ PHQ-9 | | | |
| 0.006 | 0.000 | 0.005 | 0.007 |
| CAs→ PCL-6→ PHQ-9 | | | |
| 0.249 | 0.004 | 0.242 | 0.256 |
| CAs→ SLEs → PCL-6→ PHQ-9 | | | |
| 0.008 | 0.000 | 0.007 | 0.009 |
| Total mediation effects | | | |
| 0.350 | 0.006 | 0.339 | 0.361 |
| Direct effect of CAs on PHQ-9 | | | |
| 0.087 | 0.005 | 0.078 | 0.097 |
| Dependent variable: GAD-7 | | | |
| CAs→ SLEs→GAD-7 | | | |
| 0.002 | 0.000 | 0.002 | 0.003 |
| CAs→ PCL-6→GAD-7 | | | |
| 0.231 | 0.003 | 0.225 | 0.237 |
| CAs→ SLEs → PCL-6→GAD-7 | | | |
| 0.007 | 0.000 | 0.007 | 0.008 |
| Total mediation effects | | | |
| 0.286 | 0.005 | 0.276 | 0.295 |
| Direct effect of CAs on GAD-7 | | | |
| 0.044 | 0.004 | 0.037 | 0.052 |

*Note: CAs: childhood adversities; SLEs: stressful life events; PHQ-9: the 9-item Patient Health Questionnaire, in this table it refers to the total score of PHQ-9; GAD-7: the 7-item General Anxiety Disorder, in this table it refers to the total score of GAD-7; PCL-6: the 6-item PTSD checklist, , in this table it refers to the total score of PCL-6*

**Figure S1. The correlation figure**


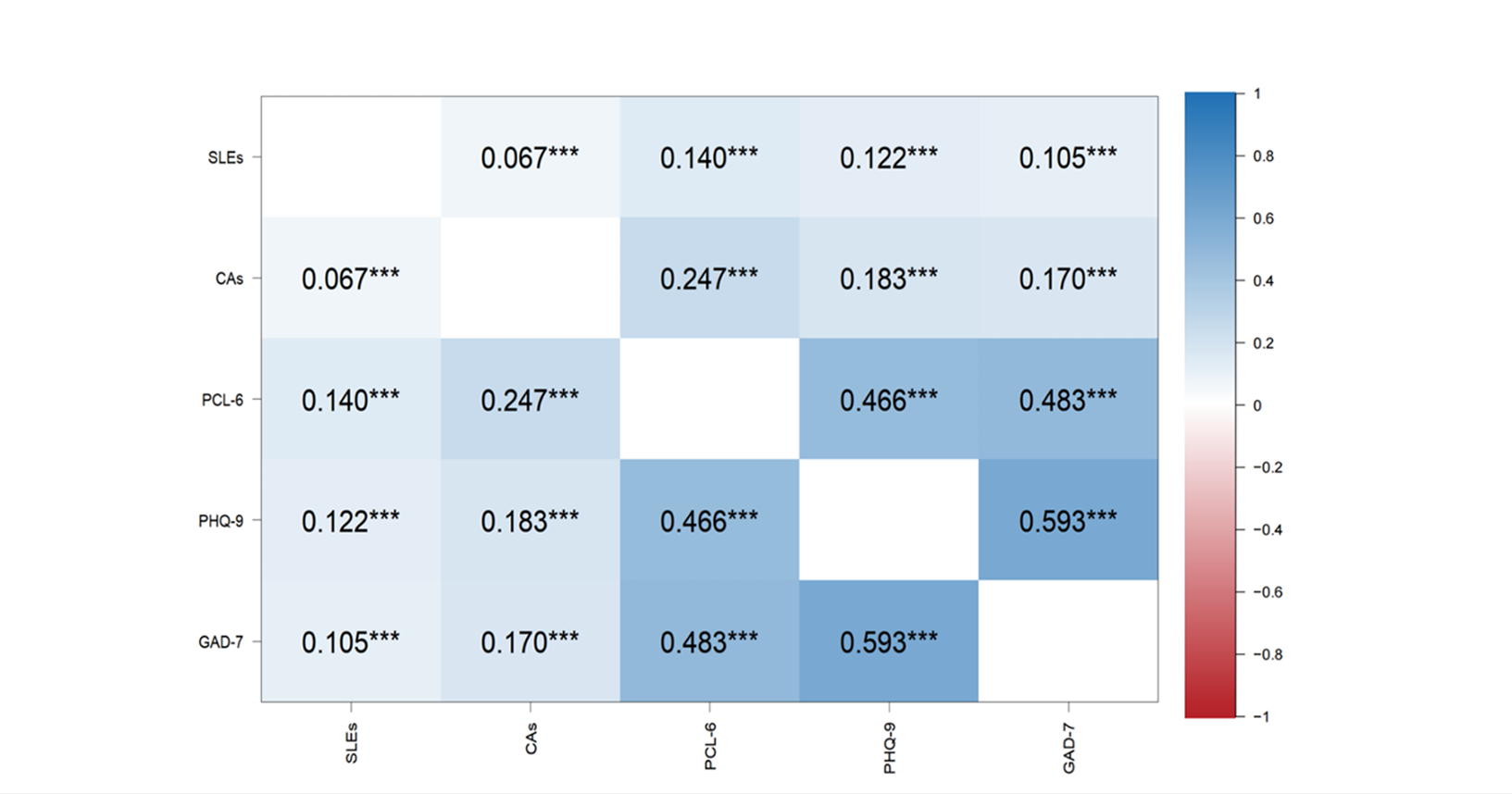


*Note: CAs: childhood adversities; GAD-7: the 7-item General Anxiety Disorder; PHQ-9: the 9-item Patient Health Questionnaire; PCL-6: the 6-item PTSD checklist; SLEs: stressful life event*
